# Cirrhotic portal hypertension treated with a 5-hydroxytryptamine receptor 1A antagonist

**DOI:** 10.1515/jtim-2026-0012

**Published:** 2026-04-04

**Authors:** Changpeng Zhu, Weihua Dong, Huaqiang Liao, Yong Lin, Weifen Xie

**Affiliations:** Department of Gastroenterology, Changzheng Hospital, Naval Medical University, Shanghai, China; Department of Interventional Radiology, Changzheng Hospital, Naval Medical University, Shanghai, China

## To the editor

Portal hypertension, a life-threatening complication of cirrhosis, arises from increased intrahepatic resistance and systemic hyperdynamic circulation.^[1,2]^ While there is no cure for cirrhosis, reducing portal hypertension significantly prevents complications and improves prognosis of cirrhotic patients.^[2,3]^ Non-selective beta-blockers (NSBBs), the current first-line therapy for both primary and secondary prophylaxis of variceal hemorrhage, remain the cornerstone of clinical management.^[3]^ However, their application in certain patients is limited by unpredictable responses and potential adverse events, particularly hypotension and bradycardia.^[4]^

Notably, the portal vein (PV) has received little attention under portal hypertensive conditions despite its critical role in maintaining portosinusoidal pressure. We have identified increased contractile tension of the PV as a significant contributor to the development of portal hypertension.^[5]^ Specifically, we demonstrated that peripheral 5-hydroxytryptamine (5-HT) enhances the contractility of the PV *via* selective activation of the 5-HT receptor 1A (HTR1A) in PV smooth muscle cells (PVSMCs). Pharmacological blockade of HTR1A or genetic deletion of *Htr1a* in preclinical models effectively ameliorates portal hypertension without affecting liver fibrosis and systemic hemodynamics, highlighting HTR1A as a promising therapeutic target for portal hypertension intervention.

Alverine citrate (ALV), a synthetic derivative of papaverine, is clinically used as an antispasmodic agent to alleviate smooth muscle spasms in conditions such as irritable bowel syndrome, painful diverticular disease of the colon, and primary dysmenorrhea.^[5,6]^ ALV was previously hypothesized to reduce the visceral pronociceptive effects of 5-HT by inhibiting the agonist action on the HTR1A.^[7]^ We also demonstrated that ALV acts as an HTR1A antagonist to modulate PV contraction and thereby reduce portal pressure without systemic hemodynamic alterations in preclinical models of portal hypertension.^[5]^ This distinct pharmacodynamic profile positions ALV as an ideal candidate for clinical translation. This report describes a proof-of-concept evaluation of ALV in two cirrhotic patients with portal hypertension.

**Figure 1:**
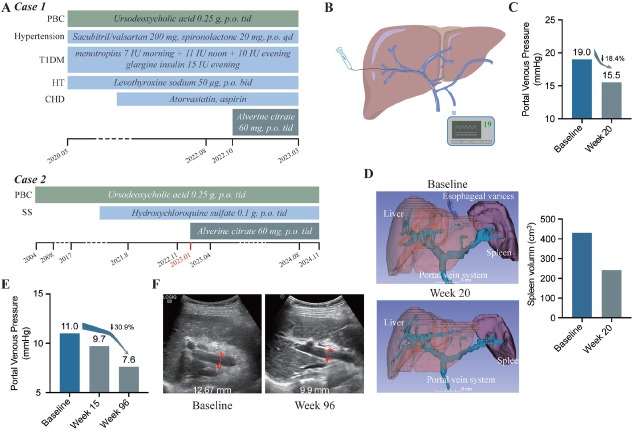
Clinical presentation and therapeutic outcomes of the patients. (A) Upper panel: Clinical timeline and pharmacotherapy for the case 1 with PBC diagnosed in 2020. The concurrent therapies included management of hypertension, T1DM, CHD, HT, and PBC. ALV was administered at a dosage of 60 mg orally, three times daily, from October 2022 through March 2023. Lower panel: Clinical timeline for the case 2 with PBC diagnosed in 2004. Sjögren’s syndrome was managed with hydroxychloroquine sulfate (0.1 g three times daily) for 13 years. ALV was administered at a dosage of 60 mg orally, three times daily, from January 2023 to November 2024. (B) Percutaneous transhepatic PVP measurement procedure. A 22-gauge Chiba needle (COOK) was percutaneously punctured into the intrahepatic portal vein under ultrasound and fluoroscopic guidance. A 0.014-inch pressure-sensing guidewire (PressureWire X, Abbott) connected to the QUANTIENTM system (St. Jude Medical, Abbott) was advanced into the main portal vein. Continuous PVP monitoring was performed after needle withdrawal. (C) Following ALV treatment, the baseline PVP was decreased from 19 mmHg to 15.5 mmHg at the 20-week assessment, representing an 18.4% reduction. (D) Left panel: Three-dimensional reconstruction of abdominal CT scans (3D Slicer software, www.slicer.org) demonstrating liver (Light brick red), spleen (muted purple), esophageal varices (periwinkle blue), and portal vein bright sky blue) morphology. Following ALV treatment, the esophageal varices were significantly alleviated, and the spleen volume was obviously reduced. Right panel: Spleen volume reduction quantified by 3D Slicer after ALV treatment. (E) The effect of long-term ALV treatment on PVP. Baseline PVP decreased from 11.0 mmHg to 9.7 mmHg at 15 weeks and 7.6 mmHg (30.9% reduction) at 96 weeks, respectively. (F) Representative ultrasound images for portal vein measurements. The portal vein width was measured at the main portal vein (indicated by double red asterisks) in the subcostal oblique plane. PBC: primary biliary cholangitis; T1DM: type 1 diabetes mellitus; CHD: coronary heart disease; HT: Hashimoto’s thyroiditis; ALV: alverine citrate; SS: Sjögren’s syndrome; PVP:portal venous pressure; CT: computed tomography.

Two female patients with primary biliary cholangitis (PBC; aged 75 and 69 years) were included in this study. Both patients progressed to cirrhosis and portal hypertension despite ursodeoxycholic acid (UDCA) therapy for 2 and 19 years, respectively (Figure 1A). The portal hypertension was evidenced by a portal venous pressure (PVP) ≥ 10 mmHg and imaging findings including portal vein dilation and splenomegaly. ALV was administered on a compassionate-use basis as an exploratory extension of a registered trial (NCT05508633) to explore its potential hemodynamic and clinical effects, given the compelling preclinical evidence. They received oral ALV (60 mg three times daily) for 20 and 96 weeks, respectively (Figure 1A). The treatment protocol received approval from the Ethics Committee of Shanghai Changzheng Hospital (2022SL050). Both patients provided written informed consent. Given ALV’s portal tropism and its direct targeting of HTR1A in PVSMCs, we prioritized PVP measurement over the hepatic venous pressure gradient (HVPG) to precisely evaluate direct pharmacodynamic responses to therapeutic intervention. PVP was measured *via* percutaneous transhepatic portal vein catheterization using a standardized protocol with a pressure-sensing guidewire (PressureWire X, Abbott) connected to the QUANTIENTM system (St. Jude Medical, Abbott), as detailed in the Supplementary Methods (Figure 1B).

Case 1: After 20-week ALV treatment, PVP decreased by 18.4% from baseline 19.0 mmHg to 15.5 mmHg (Figure 1C). Follow-up computed tomography demonstrated resolution of previously mild esophageal varices, with splenic volume decreasing from 431 cm^3^ to 242 cm^3^ (a 43.9% reduction; Figure 1D, Supplementary Figure S1A). The Child-Pugh score improved from 6 to 5 (Supplementary Figure S1B). Platelet count increased from 68 to 91 × 10^9^/L (33.8%), and serum albumin increased from 37.0 to 39.1 g/ L (5.7%). Serum alkaline phosphatase, aspartate aminotransferase and gamma-glutamyl transferase decreased from 56 to 24 U/L, 69 to 36 U/L, and 84 to 32 U/L, respectively (Supplementary Table S1). The patient also reported enhanced general well-being and health-related quality of life (HRQoL) as determined by EQ-5D-3L questionnaire (Supplementary Figure S1C, Supplementary Table S2).

Case 2: ALV treatment induced a progressive reduction in PVP, decreasing from 11.0 mmHg at baseline to 9.7 mmHg at week 15 (-11.8%) and subsequently to 7.6 mmHg by week 96 (-30.9%; Figure 1E). The PV width decreased from 12.67 mm to 9.9 mm (-21.9%) at 96-week follow-up (Figure 1F). Platelet count increased from 63 to 85 × 10^9^/L (34.9%), and serum albumin levels increased from 33.1 to 42 g/ L (26.9%; Supplementary Table S1). Treatment was associated with pruritus/fatigue alleviation, sustained HRQoL improvement (Supplementary Figure S1C, Supplementary Table S2), and a stable Child-Pugh score (Supplementary Figure S1B).

Throughout the therapeutic course, both patients maintained stable blood pressure and heart rate (Supplementary Table S3) with no adverse events or episodes of bleeding or other decompensation events, consistent with findings from our prior animal findings.^[5]^

In patients with PBC and clinically significant portal hypertension, long-term prognosis remains unfavorable despite achieving a biochemical response to UDCA treatment.^[8]^ In this study, both patients progressed to cirrhosis and developed portal hypertension despite prolonged UDCA therapy. ALV treatment subsequently resulted in reductions in PVP, accompanied by encouraging trends towards partial restoration of liver function parameters, and prevention of decompensation events. Notably, one patient exhibited marked improvement in esophageal varices and a substantial reduction in spleen volume. Importantly, extended ALV administration in these two patients was well-tolerated, with no reported side effects or drug-related adverse events. In contrast to first-line NSBBs, ALV demonstrated selective portal pressure reduction in these cases without compromising systemic hemodynamics. Throughout the follow-up period, we observed no instances of hypotension or bradycardia. As a proof-of-concept study, our findings suggest that ALV, a clinically available agent, represents a promising therapeutic candidate for portal hypertension and introduces a novel treatment paradigm by specifically targeting HTR1A in the PV.

Despite HVPG’s established role as the gold standard for evaluating portal hypertension and predicting clinical outcomes, its indirect nature means it may not accurately reflect true portal pressure under certain conditions, particularly in the presence of intrahepatic shunts.^[3]^ In contrast, direct PVP monitoring precisely targets the portal vascular compartment where ALV exerts its effect, providing a direct readout of drug-target engagement and pharmacodynamic efficacy. The reliability of PVP is supported by recent studies demonstrating its excellent correlation with standard measures and its accuracy in contexts where HVPG may underestimate true portal pressure.^[9,10]^

This study has several limitations. First, the small sample size and inclusion of only female PBC patients limit the generalizability of the findings to male patients or those with other etiologies of cirrhosis. Second, the lack of a control group makes it impossible to definitively attribute the observed improvements to ALV, as natural diseases fluctuations or placebo effects cannot be ruled out. Third, the heterogeneity in treatment duration (20 *vs*. 96 weeks) complicates direct comparison between the two cases. Fourth, the open-label design introduces potential for observation bias. Finally, the use of PVP rather than HVPG as the primary hemodynamic endpoint, while mechanistically justified, is a departure from the gold standard.

In conclusion, this proof-of-concept study suggest that ALV, a clinically available agent, represents a promising therapeutic candidate for portal hypertension and introduces a novel treatment paradigm by specifically targeting HTR1A in the PV. Well-designed prospective clinical trials are warranted to confirm the therapeutic efficacy of ALV in portal hypertension. Two multicenter, prospective clinical trials (NCT06470386, NCT06473493) are on going to further ascertain the effects and safety of ALV on portal pressure and the incidence of decompensation events in patients with cirrhotic portal hypertension.

## Supplementary Material

Supplementary Material Details
