# Supplementary material for: Cirrhotic portal hypertension treated with a 5-hydroxytryptamine receptor 1A antagonist
Source: J Transl Int Med. 2026 Apr 4;14(3):481–4. doi: 10.1515/jtim-2026-0012 (PMC13320527; doi:10.1515/jtim-2026-0012)
Supplement: Supplementary file 1 — Supplementary Material Details [file jtim-2026-0012_sm.pdf]

Supplementary Materials

Supplementary Methods

Study patients

The two patients included in this concept-of-proof study were part of an exploratory extension of a registered trial (NCT05508633). They met the following inclusion and exclusion criteria:

Inclusion criteria:

1. Age 18-75 years.

2. Confirmed cirrhosis, as diagnosed by liver biopsy or imaging findings indicative of a nodular liver, splenomegaly and/or collateral.

3. Portal venous pressure (PVP) ⩾ 10 mmHg.

4. Signed informed consent.

Exclusion criteria:

1. Use of non-selective beta-blockers (*e.g.*, carvedilol, propranolol) or statins within 1 month prior to dosing.

2. Presence of moderate or massive ascites, overt hepatic encephalopathy, gastrointestinal bleeding and other complications within one week prior to enrollment.

3. History of splenectomy, cardia periesophageal vascular dissection, transjugular intrahepatic portosystemic shunt (TIPS), liver transplantation, *etc*.

4. Coagulopathy, including platelet count < 50×10^9/L, international normalized ratio (INR) of prothrombin time ⩾ 1.5.

5. Serum total bilirubin ⩾ 5 fold of upper limits of normal; serum sodium level < 125 mmol/L; white blood cell count < 1×10^9/L.

6. Severe chronic renal insufficiency (eGFR [CKD-EPI] < 20 mL/min/1.73 m^2).

7. Presence of hepatic vein, portal vein, splenic vein thrombosis or cavernous transformation of the portal vein.

8. Poorly controllable hypertension or diabetic patient; severe background disease like chronic respiratory failure, circulatory failure, kidney failure, *etc*.

9. Clinically diagnosed or suspected as malignancy, including hepatocellular carcinoma.

10. Any uncontrolled active infection (*e.g.*, lung infection, abdominal infection, HIV, *etc.*) 4 weeks prior to enter in the study.

11. Patient who are allergy to the experimental drug.

12. Patients with abnormal mental symptoms or taking tricyclic antidepressants and similar drugs in the past 4 weeks.

13. Gestation or lactation period women and women who plan to get pregnant during the study period.

14. Patients who are participating other trials or have taken part in other in the past 4 weeks.

15. Other situation where principal investigator (PI) thinks the patient should be excluded.

Further details of the trial (NCT05508633) are available at https://clinicaltrials.gov.

Percutaneous portal vein puncture and direct PVP measurement

While operator blinding was not feasible in this open-label pilot study, all PVP measurements were conducted by the same experienced interventional radiologist to maintain consistency. After fasting for more than 6 hours, patients underwent non-invasive vital sign monitoring, including electrocardiography, arterial blood pressure, and pulse oximetry. Local anesthesia with lidocaine was administrated before the puncture. Under ultrasound guidance, a 22-gauge

Chiba needle (COOK) was percutaneously punctured into the intrahepatic portal vein, and correct positioning was confirmed by and fluoroscopic imaging. A 0.014-inch pressure-sensing guidewire (PressureWire X, Abbott), which was wirelessly connected with the QUANTIENTM Measurement System (St. Jude Medical, Abbott) and previously zeroed in 37°C sterile saline, was inserted into the Chiba needle and pushed smoothly into the main portal vein based on the orientation of the vessel under fluoroscopic visualization. The needle was then withdrawn, while the guidewire was left in place and secured to the chest wall with adhesive tape. Then, portal venous pressure was continuously monitored and recorded.

Supplementary Figures and Tables

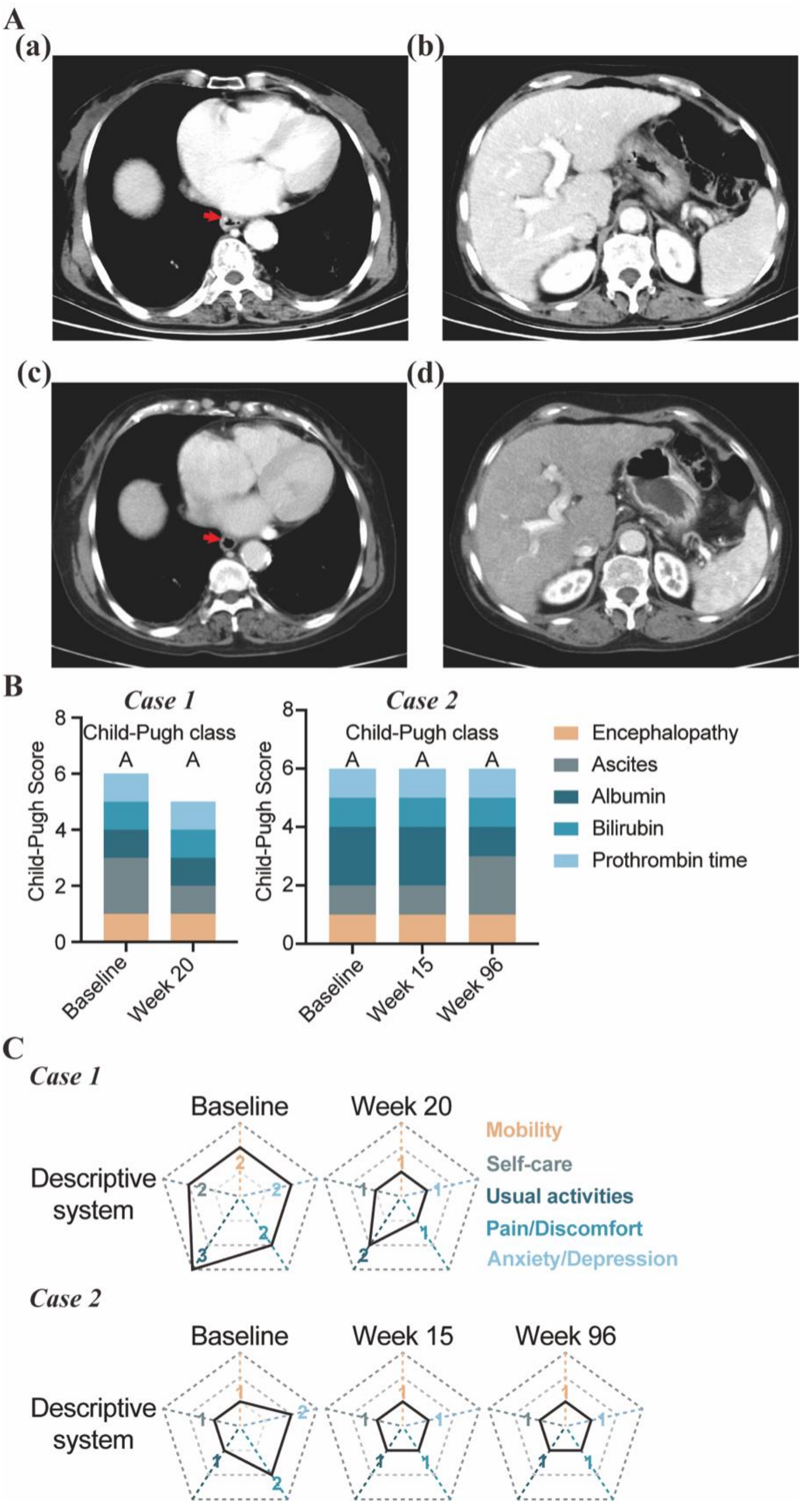

Supplementary Figure S1: Representative portovenous phase abdominal enhanced computed tomography images of case 1. (A) (a) and (b) Baseline abdominal CT images. Panel a reveals prominent esophageal varices (indicated by the red arrow), while Panel b displays an enlarged portal vein and spleen. (c) and (d) Abdominal CT images at 20 weeks post-treatment

with ALV. Panel c shows a significant improvement in esophageal varices (indicated by the red arrow). (B) Child-Pugh score improvement in case 1 from 6 to 5 points, primarily due to ascites alleviation following ALV administration. Child-Pugh score was stable in case 2. (C) HRQoL assessed by the EQ-5D-3L questionnaire. Scores across five domains (mobility, self-care, usual activities, pain/discomfort, anxiety/depression) improved at 20 weeks in case 1 (upper panel). Scores demonstrated improvement at 15 weeks and remained stable up to week 96 post-treatment in case 2 (lower panel). CT: computed tomography; ALV: alverine citrate; HRQoL: health-related quality of life.

Supplementary Table S1: Laboratory investigations.

Variable	Case 1		Case 2			Reference range
	Baseline	Week 20	Baseline	Week 15	Week 96	
**Total white blood cell count ($\times10^9$/L)**	3.8	4.9	2.9	4.4	3.3	3.5-9.5
**Total red blood cell count**	4.20	4.68	3.72	3.63	3.40	3.80-5.10

**(×10^9/L)**						
**Hemoglobin (g/L)**	126	144	121	120	119	115-150
**Platelet count (×10^9/L)**	68	91	63	43	85	125-350
**Total bilirubin (μmol/L)**	19.4	19.7	19.4	11.9	9.5	3.4-17.1
**Direct bilirubin (μmol/L)**	3.4	2.8	3.6	2.4	1.6	0-3.4
**Serum albumin (g/L)**	37.0	39.1	33.1	33.6	42.0	40.0-55.0
**Alanine transaminase (U/L)**	56	24	10	12	15	7-40
**Aspartate aminotransferase (U/L)**	69	36	20	28	27	13-35

**Alkaline phosphatase (U/L)**	135	65	70	70	101	35-135
**Gamma-glutamyl transferase (U/L)**	84	32	11	10	11	7-45
**Serum creatinine (μmol/L)**	55	68	68	70	101	41-81
**Serum total cholesterol (mmol/L)**	4.12	NM	3.81	3.10	3.48	< 5.20
**Triglycerides (mmol/L)**	1.11	NM	1.08	0.73	0.76	0-1.70
**Prothrombin time (S)**	14.8	14.3	14.6	15.9	13.1	11.0-13.0
**International normalized ratio**	1.18	1.13	1.05	1.33	1.08	0.79-1.14

**Alpha-fetoprotein (μg/L)**	3.97	3.45	1.53	2.04	1.64	0-20.00
**CEA (μg/L)**	4.43	4.99	1.34	2.32	1.89	0-5.00
**CA19-9 (U/mL)**	52.08	47.87	9.15	10.78	13.5	0-39.00
**CA125 (U/mL)**	12.4	12.67	14.78	15.74	13.5	0-35.00

Supplementary Table S2: HRQoL assessed by EQ-5D-3L.

Variable*	Case 1		Case 2		
	Baseline	Week 20	Baseline	Week 15	Week 96
**Mobility**	2	1	1	1	1
**Self-care**	2	1	1	1	1
**Usual activities**	3	2	1	1	1
**Pain/discomfort**	2	1	2	1	1
**Anxiety/ depression**	2	1	2	1	1

**EQ visual analog scale**	30	85	70	85	90

*Scores across five domains (mobility, self-care, usual activities, pain/discomfort, anxiety/depression) and visual analog scale (VAS; 0–100). HRQoL: Health-related quality of life.

Supplementary Table S3: Systemic hemodynamic measurements at each follow-up visit.

Variable*	Case 1		Case 2		
	Baseline	Week 20	Baseline	Week 15	Week 96
**Systolic blood pressure (mmHg)**	132	130	122	122	130
**Diastolic blood pressure (mmHg)**	84	80	80	80	84
**Heart rate (beats/min)**	62	70	72	73	75

[*]Data were collected each morning after admission during follow-up hospitalizations.
